# Frequency of fokI and taqI polymorphism of vitamin D receptor gene in Indian population and its association with 25-hydroxyvitamin D levels

**DOI:** 10.4103/0971-6866.60186

**Published:** 2009

**Authors:** Aparna A. Bhanushali, Namrata Lajpal, Smita S. Kulkarni, Sandeep S. Chavan, Sarita S. Bagadi, Bibhu R. Das

**Affiliations:** Research and Development, SRL Ranbaxy Ltd, Plot No. 124, MIDC,17^th^ Street, Andheri (East), Mumbai - 400 093, India

**Keywords:** FokI, population genetics, single nucleotide polymorphism, TaqI, vitamin D receptor, 25-hydroxyvitamin D

## Abstract

**BACKGROUND:**

The VDR protein is at the centre of the vitamin D endocrine system, a complex physiological system with substantial feedback regulatory mechanisms involved in maintaining serum calcium and 1, 25 dihydroxy vitamin D3. Variations in VDR gene are shown to have implications in several diseases and have also been implicated as an important genetic factor affecting bone mass.

**AIM:**

To determine the frequency of Fok I and Taq I variants in healthy Indian individuals and its association with 25-OH-Vitamin D levels.

**SETTINGS AND DESIGN:**

Blood samples were collected from 143 unrelated normal individuals (Male-84 and Female-59) and their genotypes determined.

**MATERIALS AND METHODS:**

After amplification by polymerase chain reaction, each polymorphism was genotyped by restriction fragment length polymorphism. For 100 normal healthy individuals 25-hydroxyvitamin D estimation was done using DiaSorin kit method.

**STATISTICAL ANALYSIS:**

Graph pad software was used to calculate the *P* values from the Chi-square.

**RESULTS:**

Out of 143 samples analyzed for FokI and TaqI polymorphisms the following genotypic frequency was obtained FF 59%, Ff 36%, ff 5% and TT 49%, Tt 43%, tt 8% respectively.

**CONCLUSIONS:**

Results indicate that the distribution of the polymorphic loci Fok I and Taq I vary considerably not only in different populations, but also within India. Furthermore, when the genotypes were analyzed with respect to 25-OH-Vitamin D levels, a significant association was seen for the Taq 1 SNP but not with the Fok I.

## Introduction

Vitamin D, is an important dietary factor that mediates its action in the body through Vitamin D receptor (VDR), a member of nuclear hormone receptor super family that modulates the transcription of target genes which help in calcium uptake or bone formation like calcium binding proteins and osteocalcin.[1-3] The gene encoding the VDR is located on chromosome 12cen-q12,[4] contains 11 exons,[5] and spans approximately 75 kilobases of genomic DNA.[6] VDR gene has also been suggested as one of the candidate genes for genetic control of bone mass. Allelic variants of the gene encoding VDR, recognized by *Apa*I (allele A/a), *Bsm*I (allele B/b), *FokI* (allele F/f) and *TaqI* (allele T/t) restriction endonucleases, have been associated with Bone Mass Density (BMD)[7-10] in many studies as well as with bone loss in elderly subjects[11,12] and gain after 1, 25-dihydroxy Vitamin D3 treatment. The FokI polymorphism is a T/C transition polymorphism (ATG to ACG) at the first of two potential translation initiation sites in exon II[13] has been defined using the FokI restriction endonuclease.[14] The TaqI polymorphism is a T/C nucleotide substitution (ATT to ATC) leading to a synonymous change at codon 352 (isoleucine) in exon IX[15] Bsm I[8] and ApaI[16] restriction site polymorphisms occur in the intron separating exons VIII and IX. A strong concordance exists between the absence of the BsmI (B allele) and presence of the TaqI (t allele) sites,[8] and these sites show significant linkage disequilibrium with the ApaI polymorphism. However, the agreement on this relationship is not universal. The discrepancies between studies addressing genetic risks may be attributed to genetic heterogeneity, population admixture and gene-environment or gene-gene interactions.[17,18] Numerous reports are available on VDR SNP's with respect to different diseases world wide, however, reports from India are few and hence, in this study we present the frequency of FokI and TaqI polymorphisms in normal healthy Indian population and their association with the 25-OH-Vitamin D levels.

## Materials and Methods

### Subjects

Blood samples were collected from 143 unrelated normal individuals (Male-84 and Female-59) with informed consent. Individuals included were healthy adults between 25-60 years of age presenting for routine health check-up. The individuals had to fill up a detailed questionnaire regarding, medical history, with specific emphasis on fractures and as well as any family history of the same. Blood specimens were collected after an overnight fast of 12 hours by veni-puncture using the vacutainer system from Becton Dickinson (Franklin Lakes, NJ, USA) in the anti-coagulant EDTA as well as plain bulb for serum.

### DNA extraction

The genomic DNA was isolated from peripheral blood using QIAamp^®^ DNA Blood Mini Kit (Qiagen, Hilden, Germany). DNA yield and purity was determined by measuring absorbance at 260/280 nm.

### Vitamin D estimation

For 100 normal healthy individuals 25-hydroxyvitamin D estimation was done using DiaSorin kit method (DiaSorin, Stillwater, Minnesota USA)

The DiaSorin 25-OH-D estimation consists of a two-step procedure, the first one involves a rapid extraction of 25-OH-D and other hydroxylated metabolites from serum with acetonitrile. Treated sample is then assayed using an equilibrium RIA procedure. The RIA method is based on an antibody with specificity to 25-OH-D.

### Polymerase chain reaction

Reaction mixtures of 50 µl were used in PCR for the VDR gene (FokI, TaqI) polymorphism and DNA samples were amplified in MJ Research Peltier Thermal Cycler (PTC-200). Gels were visualized under UV transilluminator imaging system. The primers used were as reported earlier for FokI[19] and for TaqI.[20]

## PCR Cycling Conditions

### FokI polymorphism

**Figure 1:**
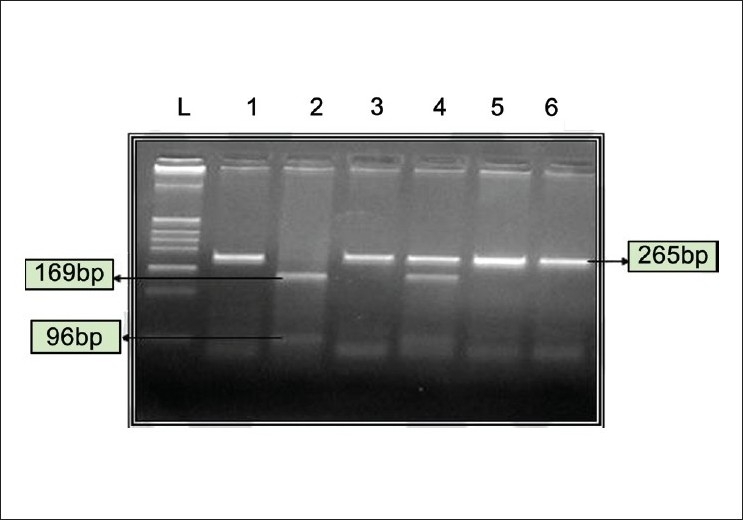
Restriction Endonuclease digestion for FokI polymorphism. FF indicates absence of FokI RE site, ff-presence of FokI RE site. L-DNA ladder pBR322 digested with Hinf1. Lane 1- Undigested, Lane 2- ff (169 bp and 96 bp), Lanes 3, 5 and 6-FF (265 bp), Lane 4-Ff (265 bp, 169 bp and 96 bp)

DNA samples were amplified with cycling parameters as follows: Initial denaturation at 94°C for 5 minutes followed by 32 cycle of 94°C for 45 seconds, 58°C for 45 seconds, followed by 72°C for 45 seconds, and a final extension at 72°C for 7 minutes. The reaction mixture consisted of 100-200 ng genomic DNA, 200 mM each of dATP, dCTP, dGTP, dTTP (Sigma, USA), 5 µl of 10 X PCR Buffer, 0.3 µl of 1.5 U of Taq DNA Polymerase (Sigma, Missouri, USA), 20 pM of each primer (Sigma, Missouri, USA). Following amplification, the translation initiation site of the VDR gene was detected by RFLP (Restriction Fragment Length Polymorphism) using the restriction endonuclease *Fok1* (New England Biolabs) at 37°C for 4 hours. Digested restriction fragments were separated on 2.5% (w/v) agarose (Sigma) gels. Bands were visualized on an UV Transilluminator Imaging system. Depending on the digestion pattern, genotypes were assigned as follows: FF homozygous for the absence of the FokI site with an undigested 265 bp band; ff homozygous for the presence of the FokI site with complete digestion into 169 bp and 96 bp bands and Ff in case of heterozygosity all three bands (265 bp, 169 bp and 96 bp) were observed [Figure 1].

### TaqI polymorphism

The PCR conditions were - initial denaturation at 94°C for six minutes followed by 35 cycle of 94°C for 45 seconds, 63°C for 60 seconds, followed by 72°C for 75 seconds, and a final extension at 72°C for seven minutes.

**Figure 2:**
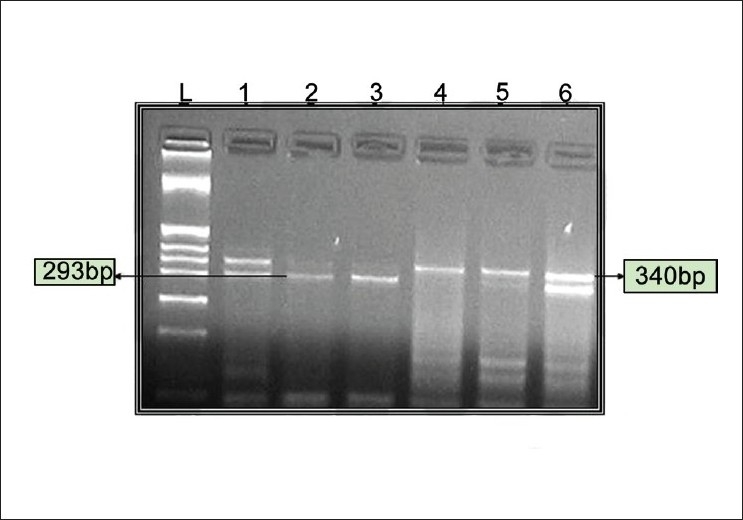
Restriction Endonuclease digestion for TaqI polymorphism. TT indicates absence of Taq1 RE site, tt-presence of Taq1 RE site. L-DNA ladder pBR322 digested with Hinf1. Lanes 1and 6 tt (340 bp, 293 bp and 47 bp), Lanes 2 and 3- Tt (293 bp and 47 bp), Lanes 4 and 5 TT (340 bp)

Following amplification the site on VDR gene was detected by RFLP (Restriction Fragment Length Polymorphism) using the restriction endonuclease *Taq1* (GENEI, Banglore, INDIA) at 65°C for four hours. Digested restriction fragments were separated on 2.5% (w/v) agarose (Sigma) gels. Bands were visualized on an UV Transilluminator Imaging system. Genotypes were assigned as follows: TT homozygous for the absence of TaqI site 340 bp only; tt homozygous for the presence of TaqI site 293 bp and 47 bp, in case of heterozygosity Tt, all three bands (340 bp, 293 bp and 47 bp) were exhibited [Figure 2].

### Sequencing

Genotyping of 10% of samples was confirmed by sequencing [Figure 3a and b]. Amplified products were purified using QIAquick PCR purification kit (Qiagen, Hilden, Germany) and directly sequenced to identify the polymorphic site by Automated ABI prism 3100 *Avant* Genetic Analyzer (Applied Biosystems Inc., Foster city, Calif.) using ABI prism BigDye terminator kit (version 3.1).

**Figure 3a:**
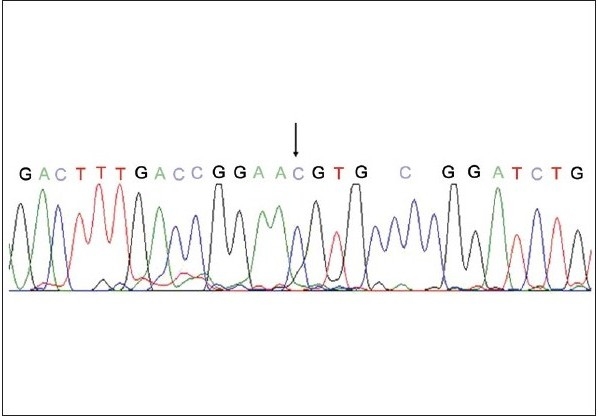
Sequencing to confirm the genotyping results; Sequencing result of the FokI polymorphism, arrow indicates the presence of the minor allele C at the polymorphic site instead of T

**Figure 3b:**
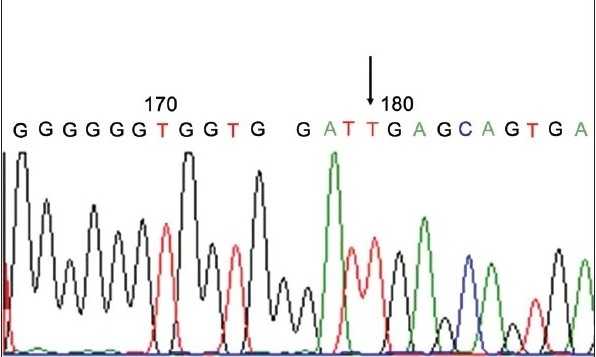
Sequencing to confirm the genotyping results; Sequencing result of the TaqI polymorphism, arrow indicates the presence of the major allele T

### Statistical analysis

Allele frequency was calculated as the number of occurrences of the test allele in the population divided by the total number of alleles. Hardy Weinberg Equilibrium was also applied to the allelic frequencies. Chi-square test was applied to compare the allelic frequency of different populations of the present study with different populations. The Chi-square test was also performed for comparison based on gender and finally also for correlation of 1, 25-dihydroxy vitamin D3 levels with the polymorphisms. Graph pad software was used to calculate the *P* values from the Chi-square. A *P* value < 0.05 is considered significant for the data at 5% level of significance.

## Results

Out of 143 samples analyzed for FokI and TaqI polymorphisms the following genotypic frequency was obtained FF 59%, Ff 36%, ff 5% and TT 49%, Tt 43%, tt 8% respectively [Table 1]. The allelic frequency was in agreement with Hardy-Weinberg equilibrium, which is an important confirmation in studies involving two alleles in population genetics. We tried to estimate the genotypic distribution of FokI and TaqI based on gender, although males showed high frequency for FF and TT genotype in comparison to females (64% vs 52.5% and 51% vs 45% respectively), a significant difference was not observed on application of Chi-square test [Table 2]. Upon comparison of FokI [Table 3] and TaqI [Table 4] frequencies of different populations, including previous study from India with the present study by using χ^2^ test, a significant difference was observed.

**Table 1:** Genotypes and allele frequency distribution of vitamin D receptor gene polymorphism in Indians

Polymorphism	Genotypes % (n = 143)			Allele frequencies	
Fok1	FF	Ff	ff	F	f
	59 (85)	36 (51)	5 (7)	0.77	0.22
Taq1	TT	Tt	tt	T	t
	49(70)	43(62)	8(11)	0.70	0.29

**Table 2:** Genotypes based on gender

Polymorphism	Genotypes % (n = 143)		
Fok1	FF	Ff	ff
Males	64.2 (54)	32.3 (27)	3.5 (3)
Females	52.6 (31)	40.6 (24)	6.8 (4)
Taq1	TT	Tt	tt
Males	51.1 (43)	40.5 (34)	8.4 (7)
Females	45.8 (27)	47.5 (28)	6.7 (4)

**Table 3:** Genotype frequency distribution of vitamin D receptor Fok1 gene polymorphism in various populations and Chi-square values

Population (ref)	Sample size	FF %	Ff %	ff %	χ^2^$	*P* values#
Caucasians UK[30]	108	48	41	11	6.40	0.04
French[31]	100	43	47	10	11.03	0.004
Japanese[21]	249	37	51	12	19.76	0.0001
North Indians[22]	346	44	49	7	9.731	0.007
Present study	143	59	36	5		

Each population has been compared w.r.t. the Indian population and the Chi-square and *P* values calculated.

$ :- Χ^2^ tabulated at 5% level of significance (i.e.0.05) and 2 degrees of freedom.

# :- *P* values have been calculated from χ^2^ using Graph Pad Prism software

**Table 4:** Genotypes and allele frequency distribution of vitamin D receptor Taq1 gene polymorphism in various populations and Chi-square values

Population^*^ (ref)	Sample size	FF %	Ff %	ff %	χ^2^$	*P* values#
French[31]	189	33	49	18	13.21	0.0014
Japanese[32]	488	77	22	1	68.182	0.0001
Chinese[33]	144	90	10	0	59.23	0.0001
North Indians[22]	346	36	44	20	14.65	0.0007
Present study	143	48.9	44.3	7.6		

Each population has been compared w.r.t. the Indian population and the Chi-square and *P* values calculated

$ :- χ^2^ tabulated at 5% level of significance (i.e.0.05) and 2 degrees of freedom

# :- *P* values have been calculated from χ^2^ using Graph Pad Prism software

In addition to this, we have also tried to correlate levels of vitamin D_3_ with FokI and TaqI genotypes. 25-hydroxyvitamin D levels were analyzed for 100 individuals, who were segregated into low vitamin D and those falling under the normal range (9-37.6 ng/ ml). Out of 100 subjects, 34 had low vitamin D and the remaining 66 were within the normal range. No correlation was observed with respect to the FokI SNP (*P* value > 0.05 i.e.0.954), but the TaqI SNP showed a strong correlation with the 25-OH-vitamin D levels (*P* value < 0.05 i.e 0.021) [Table 5].

**Table 5:** Correlation of FokI and TaqI genotypes with the vitamin D3 levels

Genotypes	low vitamin D (> 9 ng/ml) % (n)	within normal range (9-37 ng/ml) % (n)	N
FF	32.6 (17)	63.4 (35)	52
Ff	35.7 (15)	64.3 (27)	42
Ff	33.3 (2)	66.7 (4)	6
		*P* value = 0.954	N = 100
TT	27 (14)	53 (38)	52
Tt	47.6 (20)	52 (22)	42
Tt	0 (0)	100 (6)	6
		*P* value = 0.021	N = 100

## Discussion

Vitamin D function mediates its effects via the VDR which is a potent regulator of bone and calcium homeostasis as well as in immunomodulation, cellular differentiation and replication in different target tissues. VDR gene polymorphisms have been associated with multiple traits and disease phenotypes like primary hyperparathyroidism, Grave's disease, Type I-diabetes mellitus and osteoporosis.

Several polymorphisms have been identified in VDR gene viz, FokI, TaqI, BsmI and ApaI. The following genotypic frequency was obtained for the FokI FF 59%, Ff 36%, ff 5% and TaqI TT 49%, Tt 43%, tt 8% respectively in our study. The frequency of the polymorphisms is dependent on ethnicity, hence when these frequencies were compared using chi-square with other populations the analysis was found to be statistically significant for FokI and TaqI. [Tables 3 and 4]. The frequency of these polymorphisms not only vary between our population and Caucasians, but also vary from other Asian countries like Japan[21] (FF-37%, Ff-51%, ff-12% and TT-77%, Tt-22%, tt-1% respectively). The frequency of the FokI and TaqI genotypes in the present study also shows different results than that of a study conducted in North Indian population (FF-44%, Ff- 49%, ff-7% and TT-49%, Tt-40%, tt-11% respectively).[22] Most data indicate that the F allele is more effective than the f allele in trans-activation of the 1, 25(OH)2-D3 signal.[23,24] Indians have lower bone density as compared to the North American and European counterparts,[25-27] and differences in the frequency of genetic variants may be a contributing factor. Thus the current data signifies an impact of ethnicity and provides a basis for future epidemiological and clinical studies.

Another important aspect of the study was the correlation between FokI, TaqI SNP's and 25-OH-vitamin D levels [Table 5]. The TaqI SNP showed a strong correlation with respect to the Vitamin D_3_ levels (*P* = 0.021) whereas no correlation was observed with respect to FokI SNP (*P* > 0.05) upon chi-square test analysis. The TaqI SNP is reported to be in linkage disequilibrium with BsmI and ApaI and can be considered as a marker for bone mineral density in individuals. Bone density was observed to be higher in some[28] but not all studies[29] in subjects with the bbaaTT haplotype than in those with BBAAtt haplotype (The VDR alleles were classified according to the presence (b, t, a) or absence (B, T, A) of the BsmI, TaqI and ApaI restriction enzyme cutting sites). The possible explanation for association of the Taq1 genotype with Vitamin D levels could be that polymorphisms in the VDR gene are known to influence calcium metabolism, which in turn plays an important role in feedback mechanism of Vitamin D levels. Alternatively it is also possible that the TaqI polymorphism may be in linkage dis-equilibruim with another marker that may be the true causative factor influencing the Vitamin D levels.

## Conclusion

We have determined the frequency of FokI and TaqI polymorphism in the VDR gene in the Indian population. Also, there is association of the Taq1 genotypes with the Vitamin D3 levels.
